# Online short videos promoting public breast cancer literacy: a pretest-posttest control group trial on efficiency, attitude, and influencing factors

**DOI:** 10.3389/fpubh.2023.1198780

**Published:** 2023-06-15

**Authors:** Qian-Rui Xu, Pei-Zhu Wu, Jia-Zi Du, Wen-Jun Zhuang, Xiao-Tong He, Yong-Yong Ma, De Zeng, Yuan-Ke Liang, Xiao-Yang Xu, Lei Xie, Hao-Yu Lin

**Affiliations:** ^1^Department of Thyroid and Breast Surgery, Clinical Research Center, The First Affiliated Hospital of Shantou University Medical College, Shantou, China; ^2^Cheung Kong School of Journalism and Communication, Shantou University, Shantou, China; ^3^Department of Medical Oncology, Cancer Hospital of Shantou University Medical College, Shantou, China; ^4^Department of Radiology, The First Affiliated Hospital of Shantou University Medical College, Shantou, China

**Keywords:** cancer health education, online short video, breast cancer, e-health, health literacy

## Abstract

**Background:**

Short videos on social media are playing an increasingly important role in cancer health education today. It is important to explore how the actual communication effect of health videos and the knowledge absorption of users are influenced by different factors of the video creation process.

**Objective:**

The objective of our study is to access the factors influencing breast cancer health education through short videos on efficiency and quality.

**Methods:**

Three pairs of videos about breast health were created and participants completed questionnaires before and after watching the videos. A paired *t*-test was used to analyze within-group change scores. RM-ANOVA was used to assess the relationship between the pretest, posttest, and three variables.

**Results:**

Watching short videos can significantly increase viewers’ knowledge of related health topics (*p* < 0.05). The viewers’ concentration level while watching was significantly higher for the video with background music (BGM) than for the video without BGM (*p* = 0.006). The viewers’ willingness to share was significantly higher for the video with a progress bar than for the video without a progress bar (*p* = 0.02). Using an interpreter wearing a doctor’s uniform instead of casual wear and setting a progress bar can significantly improve the efficiency of knowledge absorption (*p* < 0.05).

**Conclusion:**

A uniformed interpreter, BGM and a progress bar are factors influencing the efficiency of short health videos. They can be applied in video making to explore better ways of promoting cancer health education in the new mobile Internet environment.

## 1. Introduction

In the past decade, the importance of social media as a means of disseminating health information has grown (1, 2). According to Omboni (3), e-health was defined as “the use of communication and information technologies (ICT) to manage patients and their health in a more efficient way.” All facets of contemporary society are being impacted by the rising use of social media (2). At the same time, in the context of the rapid development of digital technology, people’s demand for health knowledge is increasing, and the influence of science popularization videos in the field of health education and publicity is becoming increasingly significant (4, 5). As awareness of women’s health issues has increased and breast cancer is drawing more attention, producers of knowledge popularization videos have started to focus on women’s health issues.

Breast cancer is the most prevalent cancer among women and a worldwide public health problem. Global Cancer Statistics 2020 pointed out that breast cancer has surpassed lung cancer as the cancer with the highest incidence rate in the world (6). Variations in global survival rates are caused by a variety of factors, including delayed diagnosis and a lack of effective treatment (6). Research has shown that the main reasons for the high incidence rate and mortality of breast cancer in developing countries are the lack of correct understanding or knowledge of breast cancer, improper screening plans, delayed diagnosis, and insufficient medical facilities (7, 8). Early detection and health education are particularly important for the prognoses of breast cancer patients (9). Improving breast cancer prognoses and survival rates through early detection is the basis of breast cancer regulations (10).

A study showed that short health videos on social media platforms are extremely popular among older women in China. Guidance through short health videos can help women establish new health awareness and lifestyle dynamics (11). With the increasing demand for public health knowledge and the improvement of health short videos, many scholars have conducted multidimensional discussions on the current dissemination of health short videos, the problems they face, and the optimization of countermeasures (12, 13). Among them, the exploration of communication strategies mainly focuses on three aspects: short video content, interactive dialog, and platform supervision. For example, strategies for improving content include the liveliness of the theme, the scientificity of the content, and the diversity of presentation forms. Platform supervision and guidance need to be carried out from aspects such as subject review, content control, and audience acceptance to control communication risks. Previous studies have played a promoting role in creating a good health short video communication order and better playing the positive role of short video in promoting health communication. However, existing research has rarely systematically examined the impact of video production factors on communication effectiveness.

Previous research on the impact of popular science videos also discovered that seductive details refer to interesting content that is not necessarily related to the teaching objectives (12) and can be presented in various forms, such as text, illustration, dialog, and BGM. However, the message might also be interrupted by attention shift and lack of coherence. Interference with cognitive basic patterns and working memory overload will lead to the undesired effects (13). Therefore, it is important to explore how the actual communication effect of health videos and the knowledge absorption of users are influenced by different factors of the video creation process to explore better ways of promoting the communication of health videos in the new mobile Internet environment.

According to prior research on health science popularization and the perception of doctors, the attire of the characters in the video, the presentation of the video directory structure, and the background music (BGM) may be the three main variables that affect the impact of science popularization video transmission. (1) A survey found that 69.9% of the 153 patients surveyed preferred doctors to wear white coats, because it made them feel more at ease and confident in their doctors (14). Zou et al.’s (15) research shows that doctors wearing white coats are considered more professional, responsible, and knowledgeable, and have higher medical safety than those not wearing white coats. In another survey, when asked about their preferences for physician wear, respondents significantly favored professional attire with a white coat, followed by surgical scrubs, business dress, and casual dress (16). According to a study of TED videos, science videos presented by academics were more liked than those presented by nonacademic (17). (2) A progress bar with a directory function can effectively display the video network directory structure. When a progress indicator design that included the percentage of task completion was used, the quality of the user experience increased (18). A constant progress bar can keep users informed without raising their arousal or focusing their attention on temporal stimuli (19). (3) Previous research has also concentrated on the effect of BGM. Videos with BGM were found to be popular among students (20). An analysis of music videos on YouTube revealed that music could be a helpful tool for science communication and science education (21). Evidence indicated that listening to music during a colposcopy significantly reduced anxiety and discomfort (22). At the same time, according to Ma et al.’s (23)research, short music videos cause viewers to develop psychological emotions and affect their emotional responses. This indicates that BGM in short videos may significantly affect the valence of emotions and affect the effectiveness of video communication.

This study examines the factors that affect the transmission of health science short videos and discusses the impact of visual and auditory factors on the transmission effect of these videos. In the form of a questionnaire, we investigated the different effects on patients before and after watching videos to verify users’ information absorption and conversion degree of short video content. The aim was to reflect the communication effect from multiple perspectives.

## 2. Methods

### 2.1. Hypothesis

Based on the above discussion, in addition to the content of short videos, some auditory and visual forms of expression, such as background music, interpreter’s dress, program progress bar, and other factors, may affect the attention of viewers and ultimately affect the absorption efficiency of popular science knowledge. Therefore, this study proposes the following three hypotheses:

*H1*: A interpreter wearing the uniform is a factor influencing the efficiency of short health videos.

*H2*: BGM is a factor influencing the efficiency of short health videos.

*H3*: A progress bar is a factor influencing the efficiency of short health videos.

The three hypotheses put forward 3 variables, so we conducted a pretest-posttest control group trial on participants’ performance in their breast-health literacy under the 3 variables.

### 2.2. Materials and methods

**Figure 1:**
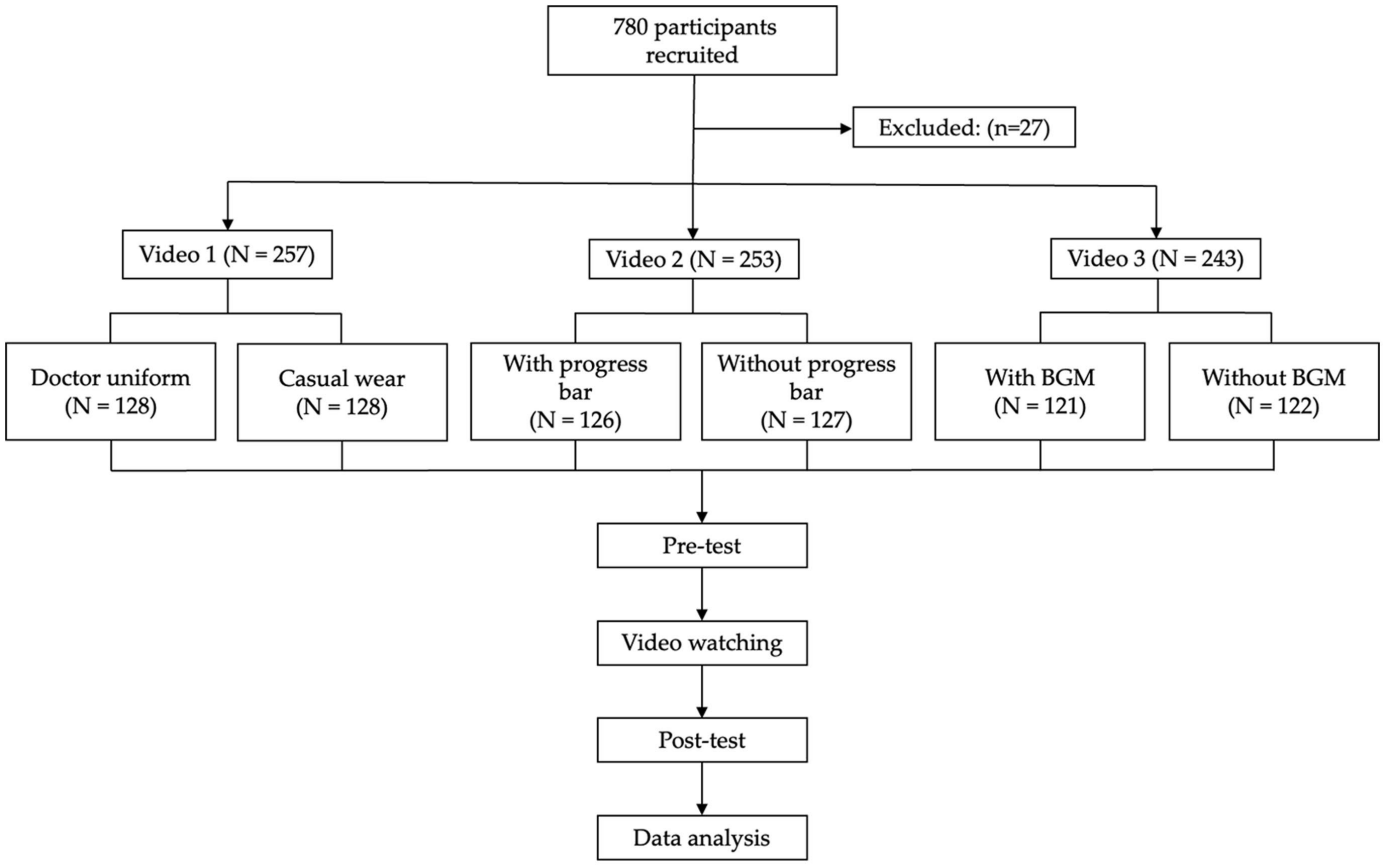
Study design.

We used a pretest-posttest control group design, which we developed on the basis of literature on the effect of online short videos. This study was conducted to assess the influence of three variables on the quality of breast health public education (Figure 1): (1) How the speaker dresses (a doctor’s white coat or casual wear); (2) the presence or absence of BGM; and (3) the presence or absence of a progress bar. The study was approved by the Ethics Committee of Shantou University Medical College, Ethical Review No. B-2022-289. A pilot study was conducted on a small sample beforehand to explore the public acceptance of the topic, BGM, and the duration of the video and to ensure that the tested videos were standardized and validated for the research.

### 2.3. Video preparation

To present the influence of the three variables mentioned above, the video production team, consisting of proficient short video producers and science popularization workers with medical backgrounds, created three groups of videos on three topics: (1) hyperplasia of mammary glands (2) Familial aggregation of breast cancer (the definition of familial clustering tendency of breast cancer and recommendations for high-risk individuals with a high incidence of breast cancer in their family); (3) marriage, childbirth, and breast cancer (how marriage, childbirth, and contraceptives affect the incidence of breast cancer by affecting estrogen). AE (Adobe after Effects) and AI (Adobe Premiere) were used to clip the video and design the background, mask, and text layers to form the progress bar. The pink mask gradually expands over time to cover the background layer, allowing viewers to visually see the progress of the video. The work was completed under the direction of a Ph.D. in sociology and a breast surgeon, who acted as gatekeepers for the video design and scientific rationality, respectively. The duration of the video was between 3 and 4 min. Except for the variables we studied, all control variables in every pair of videos were completely consistent (24).

### 2.4. Questionnaire

To measure the effectiveness of the three variables mentioned above, we developed 2 questionnaires for the participants to complete before and after watching the three pairs of videos (25). The pretest consisted of two parts: (1) participants’ cognition of and attitude toward breast diseases and (2) a pretest on breast health literacy. The posttest consisted of three parts: (1) demographic information; (2) opinions about the videos; and (3) a posttest on breast health literacy. In addition, conventional attention checks were used to identify and potentially remove responses provided by careless respondents, enhancing the validity of the questionnaire.

The questionnaires passed the verification of Credamo, which evaluated their negative effect. Eligible Credamo users could access the surveys from the platform.^1^ Only those who volunteered to participate in the survey and whose participation was recognized would acquire the questionnaires through the platform. At the beginning of the questionnaire, we emphasized our objective (academic research) and responsibility (privacy of information).

The participants’ performance in both the pretest and posttest reflected changes in their breast-health literacy, thus reflecting the effect of three variables and testing our hypotheses. Questions on both the pretest and posttest consisted of single and multiple choices. To ensure that the behavior of watching the video is related to breast health knowledge rather than changes in awareness, we designed the pretest and posttest based on the knowledge points mentioned in the video. Participants can only improve their accuracy by watching the video carefully and understanding the corresponding knowledge points, which cannot be achieved only by strengthening their awareness. The options in the two tests roughly corresponded. For a couple of options distributed in the pretest and posttest, if a participant answered the former correctly and the latter wrongly, −1 was marked, meaning that his/her breast health literacy had decreased after watching the video; if a participant answered the former incorrectly and the latter correctly, 1 was marked, meaning that his/her breast health literacy had increased after watching the video; and if a participant’s answer to both the former and the latter were correct or incorrect, 0 was marked, meaning that his/her breast health literacy had not changed significantly after watching the video.

### 2.5. Sample size

For each of the videos, participants were recruited through the Credamo platform to watch the video and fill out the questionnaires. The survey lasted from April 24 to April 29, 2022, and involved 780 participants from 31 provincial-level administrative regions (excluding Hong Kong, Macao, and Taiwan). Of the 780 respondents, 27 respondents were excluded for poor quality of the questionnaires or an abnormal amount of time spent on the questionnaires (too long or too short).

During the trial, there were 780 visits to the questionnaire website, and 753 (96.5%) participants were found to be eligible for the study. No significant differences in age, sex, education, or employment were found between the two groups for each video pair (Table 1).

**Table 1:** Summary of participant demographics.

	Video 1 (*N* = 257)			Video 2 (*N* = 253)			Video 3 (*N* = 243)		
Demographic	Doctor uniform (*N* = 128)	Casual wear (*N* = 129)	*P*	With progress bar (*N* = 126)	Without progress bar (*N* = 127)	*P*	With BGM (*N* = 121)	Without BGM (*N* = 122)	*P*
Mean age^1^	28.78	28.02	0.652	29.04	27.78	0.251	28.06	28.44	0.687
Sex^2^			0.240			0.558			0.714
Female	93 (72.7%)	85 (65.9%)		85 (67.5%)	90 (70.9%)		83 (68.6%)	81 (66.4%)	
Male	35 (27.3%)	44 (34.1%)		41 (32.5%)	37 (29.1%)		38 (31.4%)	41 (33.6%)	
Education^2^			0.743			0.385			0.302
High school or less	4 (3.1%)	5 (3.9%)		8 (6.3%)	5 (3.9%)		6 (5.0%)	3 (2.5%)	
Some college	124 (96.9%)	124 (96.1%)		118 (93.7%)	122 (96.1%)		115 (95.0%)	119 (97.5%)	
Employment^2^			0.727			0.795			1.000
Student	49 (38.3%)	52 (40.3%)		45 (35.7%)	47 (37.0%)		39 (32.2%)	39 (32.0%)	
Worker	20 (15.6%)	13 (10.1%)		15 (11.9%)	14 (11.0%)		17 (14.0%)	13.9 (24%)	
Self-employed	11 (8.6%)	16 (12.4%)		10 (7.9%)	15 (11.8%)		13 (10.7%)	13 (10.7%)	
Civil servant	17 (13.3%)	19 (14.7%)		19 (15.1%)	21 (16.5%)		16 (13.2%)	17 (13.9%)	
Unemployed	3 (2.3%)	2 (1.6%)		4 (3.2%)	2 (1.6%)		1 (0.8%)	1 (0.8%)	
Company employee	28 (21.9%)	27 (20.9%)		32 (25.4%)	28 (22.0%)		35 (28.9%)	35 (28.7%)	

1 Analyzed by the independent-samples *t*-test.

2 Analyzed by the Chi-square test*.*

### 2.6. Statistical analysis

Descriptive statistics were calculated for the participants’ demographic data. A paired *t*-test was used to analyse within-group scores changes. RM-ANOVAs were used to assess the relationship between the pretest, posttest, and three variables, with a significance level of *p* < 0.05. Analyses were performed using SPSS software version 24.0.

## 3. Results

### 3.1. Public attitude toward health educational videos

A 5-point scale was used to evaluate the public attitude toward the videos. A score of 3 points indicated a neutral attitude. The average score was calculated and is displayed in Table 2. The viewers gave positive evaluations by responding with high points to questions about their interest in the topic, such as degree, concentration level, self-accessed understanding level, trust, and willingness to share and practice the knowledge in their lives. This proved the quality and public acceptance of these videos made for the experiment. The negative emotion brought to the viewers by the video about possible health problems was controlled and received an average score between 2.488 and 2.664. The viewers’ concentration level while watching was significantly higher for the video with BGM than for the video without BGM (*p* = 0.006). The viewers’ willingness to share was significantly higher for the video with a progress bar than for the video without a progress bar (*p* = 0.02).

**Table 2:** Public attitude toward health education videos.

	Video 1			Video 2			Video 3		
	Doctor uniform (*N* = 128)	Casual wear (*N* = 129)	*P*	With progress bar (*N* = 126)	Without progress bar (*N* = 127)	*P*	With BGM (*N* = 121)	Without BGM (*N* = 122)	*P*
Interest in the topic	3.992	3.953	0.199	3.937	3.937	0.512	3.967	3.943	0.318
Liking degree	4.266	4.186	0.232	4.159	4.205	0.44	4.298	4.221	0.995
Concentration level while watching	4.602	4.659	0.137	4.651	4.614	0.129	4.702	4.59	0.006*
Self-accessed understanding level	4.273	4.271	0.450	4.31	4.22	0.42	4.256	4.238	0.416
Trust degree	4.523	4.473	0.072	4.492	4.409	0.157	4.421	4.549	0.285
Negative emotion	2.508	2.488	0.539	2.571	2.559	0.795	2.496	2.664	0.404
Willingness to share	4.078	3.961	0.513	3.976	3.969	0.02*	4.05	4.033	0.478
Willingness to practice knowledge in life	4.359	4.419	0.535	4.286	4.346	0.143	4.355	4.303	0.178

*Indicates significance at the *p* < 0.05 level.

### 3.2. The effect of videos on promoting public breast health literacy

To assess the effectiveness of the videos, the paired *t*-test was applied to estimate the difference between the pretest and posttest.

Generally, the pretest scores were lower than the posttest scores in each group and there was a significant difference between the pretest and posttest scores in each group (*p* < 0.05), meaning that the three groups of videos significantly improved the participants’ breast healthy literacy (Table 3).

**Table 3:** Cognition and attitude to breast diseases of participants.

	Pretest score (SD)	Posttest score (SD)	*t* (pre-post)	*P*
Wearing				
Doctor uniform	9.141 (1.645)	10.055 (1.594)	−5.947	<0.001*
Casual wear	9.171 (1.621)	9.543 (1.737)	−2.054	0.042*
Progress bar				
Yes	8.691 (1.953)	10.802 (2.558)	−9.096	<0.001*
No	8.685 (1.897)	10.197 (2.320)	−7.099	<0.001*
BGM				
Yes	8.215 (1.343)	8.868 (0.875)	−5.106	<0.001*
No	8.312 (1.273)	8.623 (1.201)	−2.688	0.008*

*Indicates significance at the *p* < 0.05 level.

Specifically, in the groups in which the interpreter was dressed differently, the score difference between the pretest (9.141 ± 1.645) and posttest (10.055 ± 1.594) was higher in the group in which the interpreter wore a doctor’s white coat (pretest: 9.171 ± 1.621, posttest: 9.543 ± 1.737) than in the group in which the interpreter wore casual wear. In the group in which BGM was present or absent, the score difference between the pretest (8.691 ± 1.953) and posttest (10.802 ± 2.558) was higher in the group in which BGM was present (pretest: 8.685 ± 1.897, posttest: 10.197 ± 2.320) than in the group in which BGM was absent. In the group in which the progress bar was present or absent, the score difference between the pretest (8.215 ± 1.343) and posttest (8.868 ± 0.875) was higher in the group in which the progress bar was present (pretest: 8.312 ± 1.273, posttest: 8.623 ± 1.201) than in the group in which the progress bar was absent.

### 3.3. Video creation influencing factors on the health education effect

These experiments, which were designed to verify the influence of clothing, the progress bar, and BGM on the video science popularization effect, all adopted a mixed design, and each experiment included an intrasubject variable and an intersubject variable. Among them, the test time (pretest/posttest) was the intrasubject variable, and the intersubject variable was the clothing worn by the characters in the video (a doctor’s white coat or casual clothes), whether the video had a progress bar, and whether the video had BGM. Repeated measures ANOVA was used to test the interaction of these two factors. The simple effect test was used when the interaction was significant to show which factor influenced the effect of video science popularization.

The simple effect analysis results are shown in Table 4. In the clothing experiment, the interaction of the two factors (test time*wearing factor) was significant (*F* = 5.196, *p* = 0.023). In the pretest stage, there was no significant difference between the test scores of the two kinds of clothing (*F* = 0.022, *p* = 0.883). In the posttest stage, the score of the white coat group was significantly higher than that of the casual wear group (*F* = 6.062, *p* = 0.014). This shows that compared with casual wear, a white coat can significantly improve subjects’ cognition of video science popularization knowledge points. In the progress bar experiment, the interaction of two factors (test time*progress bar factor) had a significant edge (*F* = 3.622, *p* = 0.058). In the pretest stage, there was no significant difference between the test scores of the two progress bar types (*F* = 0.001, *p* = 0.982). In the posttest stage, the score of the group with a progress bar was significantly higher than that of the group without a progress bar (*F* = 3.881, *p* = 0.050). This shows that the presence of a progress bar can significantly improve subjects’ cognition of video science popularization knowledge points. In the BGM experiment, the interaction of the two factors (test time*BGM factor) was significant (*F* = 3.918, *p* = 0.049). In the pretest stage, there was no significant difference between the test scores of the video with and without music (*F* = 0.331, *p* = 0.566). In the posttest results, the score of the group with BGM was higher than that of the group without BGM, but the difference was only marginally significant and did not reach a significant level (*F* = 3.292, *p* = 0.071). The results show that the progress bar had a lesser effect than the other two factors on improving the subjects’ cognition of video science popularization knowledge points.

**Table 4:** Comparison of video-related knowledge understanding by participants after video watching.

	*F*	*P*
Wearing (Doctor uniform – Casual wear)		
Pretest	0.022	0.883
Posttest	6.062	0.014*
Progress bar (Yes – No)		
Pretest	0.001	0.982
Posttest	3.881	0.050*
BGM (Yes – No)		
Pretest	0.331	0.566
Posttest	3.292	0.071

*Indicates significance at the *p* < 0.05 level.

In general, the clothing of the characters in the video and BGM had a significant impact on the cognitive improvement of the subjects’ popular science knowledge, and the progress bar had a significant edge effect.

## 4. Discussion

### 4.1. Factors influencing health video effects

Previous studies of influencing factors of health video creation have focused on three main aspects: (1) conception of the script [choices of different framing techniques, choices of specific examples and general conceptions, combinations of multiple theories to convey educational messages, etc. (26, 27)]; (2) elements shown in the video [status and gender of the interpreter, presence and enhancement of animation, etc. (28)]; and (3) the influence of the platform (27).

The effect of BGM on learning remains to be discussed; as shown in this experiment, the effects are uncertain (29). A previous study revealed a dual effect of the progress bar on participants’ perception of task difficulty and duration (30). This experiment indicated that the progress bar could be conducive to video education. Previous research has revealed that wearing a doctor’s white coat makes physicians more professional and experienced (31), thus enhancing their persuasiveness. Carl Hovland, a famous experimental psychologist and one of the founders of communication science, put forward the theory of the “credibility effect” of information sources in his persuasive communication research, that is, the credibility of information sources can have a positive impact on the persuasive effect in a short time. In this experiment, the participants who watched the video with a speaker wearing a doctor’s white coat performed better than the participants who watched the video with a speaker dressed in casual wear. The image of an interpreter wearing a “white coat” in a popular science video short film is a symbol of high credibility for the audience, which is consistent with previous research. From the perspective of communication studies, establishing a good image to win the trust of the audience is an important condition for improving communication effectiveness.

### 4.2. Current health video-creation situation and advice for professional health video creators

According to the 49th Statistical Report on China’s Internet Development released by CNNIC in December 2021, the utilization rate of short videos among Chinese netizens was 90.5%, reaching 934 million (32). The use of short videos to spread knowledge has unparalleled advantages. Compared with traditional communication methods such as text and images, short videos can transform intricate knowledge into vivid animations in a more intuitive form, and shorten the distance between the public and cultural knowledge (4, 33).

However, the spread of short videos about health science popularization is currently not a positive situation. The emergence of health educational videos conforms to the development trend of the online reading environment from entertainment to knowledge, but there are also many problems in the development process. These are manifested mainly in the difficulty in determining the qualifications of communicators and distinguishing between true and false information, the serious homogenization of health knowledge content, and the lack of scientific authority for the information presented. These issues increase the difficulty of absorbing effective content from the amount of information on the Internet (5, 34). Therefore, they hinder the popularization of health knowledge on the Internet. Research has shown that the overall quality of TikTok patient educational videos about COVID-19, dermatosis, chronic obstructive pulmonary disease, and cosmetic surgery is low, and the videos even contain obvious errors. Many similar research conclusions have been obtained in studies of TikTok and YouTube videos (5, 34, 35). Health video creators should explore better ways for viewers to absorb knowledge. Making use of various factors in video creation could lead to a more systematic and better-regulated health video market.

Content is the main body of short video production and transmission, and the impact of video production factors on the communication effect is often ignored. Content is presented in various forms in videos, such as text, illustration, dialogue, BGM, and so-called seductive details, which may have an impact on the audience’s attention shift and video coherence, thus changing the actual communication effect of health videos (12). This is consistent with Tables 2, 3 and in the research results. Factors such as the clothing worn by the interpreter and the presence of BGM in the production of short breast health videos can significantly affect the cognitive improvement of the subjects’ science knowledge, which indicates the importance of scientific video production methods in breast science popularization. In addition, communication researchers should pay attention to the factors of short video production and explore more scientific short video production methods, so that popular science short videos can better promote the knowledge absorption of the audience ensure scientific accuracy and help the spread of popular science short videos in the new mobile Internet environment.

This analysis provides insights into video creation in the future. Making good use of BGM, a progress bar, and a doctor’s white coat is recommended in the creation of breast health public educational videos.

### 4.3. Limitations

This study has some limitations. First, we directly recruited the video subjects and played relevant videos for them. This test method is insufficient for simulating the situation in which the subjects browse the video on the short video platform. Second, the number of participants was limited, meaning that subgroup analysis of their educational, cultural and economic backgrounds could not be performed. Moreover, this study was a short-term effect study and data from a long-term follow-up survey were lacking. Therefore, for future research on the impact analysis of short video transmission factors, recruiting more participants for subgroup analysis and obtaining long-term intervention data to explore the impact of popular science short videos on health literacy is necessary.

## 5. Conclusion

This study researched the impact of visual and auditory factors on the transmission effect of medical science short videos designed for science popularization in breast cancer. In the form of a questionnaire, we investigated the effect difference of participants before and after watching the videos to verify users’ information absorption and conversion degree of short video content to reflect the communication effect from multiple perspectives. We found that the viewers’ concentration level while watching was significantly higher for the video with BGM than for the video without BGM. The viewers’ willingness to share was significantly higher for the video with a progress bar than for the video without a progress bar. Having an interpreter wearing a doctor’s white coat instead of casual wear and setting a progress bar can significantly improve the efficiency of knowledge absorption. This study further discussed the issues encountered in the dissemination of short health science videos and provided a reference for building a more comprehensive evaluation system of medical science videos. We believe that this study supplements the literature on the factors of making short health science videos and promotes using social media to spread medical information.

## Data availability statement

The raw data supporting the conclusions of this article will be made available by the authors, without undue reservation.

## Ethics statement

The studies involving human participants were reviewed and approved by Ethics Committee of Shantou University Medical College. The patients/participants provided their written informed consent to participate in this study.

## Author contributions

X-YX and H-YL: conceptualization. W-JZ, X-TH, and Y-YM: methodology. DZ: formal analysis. Y-KL: investigation. LX: data curation. Q-RX, P-ZW, and J-ZD: writing—original draft preparation and project administration. X-YX, LX, and H-YL: writing—review and editing. DZ and Y-KL: supervision. Y-KL, X-YX, and H-YL: funding acquisition. All authors have read and agreed to the published version of the manuscript.

## Funding

This work was supported by grants from the Youth Innovative Talents Project of Education Department of Guangdong Province (2019WQNCX028), Philosophy and Social Science Planning Project of Guangdong Province in China (GD21YXW01), Research Start-up Funding Project of Shantou University (STF19026), the National Undergraduate Training Program for Innovation and Entrepreneurship (202110560036), Natural Science Foundation Committee of China (No. 82102948), Natural Science Foundation of Guangdong Province in China (No. 2021A1515012178, 2022A1515012623, 2023A1515010252), Interdisciplinary project of Li-Ka-Shing Foundation (No. 2020LKSFG05C) and Guangdong Science and technology Special Fund project (210715106900933). The Medical Science and Technology Research Foundation of Guangdong Province (Grants No. B2020138); Shantou Technology Bureau Science Foundation (Grants No. [2019] 106).

## Conflict of interest

The authors declare that the research was conducted in the absence of any commercial or financial relationships that could be construed as a potential conflict of interest.

## Publisher’s note

All claims expressed in this article are solely those of the authors and do not necessarily represent those of their affiliated organizations, or those of the publisher, the editors and the reviewers. Any product that may be evaluated in this article, or claim that may be made by its manufacturer, is not guaranteed or endorsed by the publisher.

## Footnotes

^1^ https://www.credamo.com/

## Abbreviations

BGM: Background music.
