# Supplementary material for: Online short videos promoting public breast cancer literacy: a pretest-posttest control group trial on efficiency, attitude, and influencing factors
Source: Front Public Health. 2023 Jun 15;11:1198780. doi: 10.3389/fpubh.2023.1198780 (PMC10310936; doi:10.3389/fpubh.2023.1198780)
Supplement: Supplementary file 1 [file Data_Sheet_1.PDF]

Questionnaire for Video 1

Thank you for participating in this survey! This anonymous survey was initiated by the "Beauty in the Bosom" video team to explore the influencing factors of science popularization through short video dissemination, to promote better science popularization activities. We are committed to creating professional and authoritative short videos on breast knowledge popularization. The team will keep your answers confidential and use them only for research and academic purposes. The questionnaire includes a pre-test, video, and post-test. Please answer truthfully and submit as instructed. Thank you for your cooperation!

Part One: This section is a survey about your knowledge and attitude towards breast diseases.

1.1. I have a good understanding of breast diseases.
Strongly disagree ( ) Disagree ( ) Neutral ( ) Agree ( ) Strongly agree ( )

1.2. I am willing to learn about breast disease-related knowledge.
Strongly disagree ( ) Disagree ( ) Neutral ( ) Agree ( ) Strongly agree ( )

1.3. I believe that watching educational videos about breast diseases helps improve awareness of one's own health.
Strongly disagree ( ) Disagree ( ) Neutral ( ) Agree ( ) Strongly agree ( )

1.4. I believe that my breast health is good.
Strongly disagree ( ) Disagree ( ) Neutral ( ) Agree ( ) Strongly agree ( )

1.5. I am concerned about developing breast cancer in the future.
Strongly disagree ( ) Disagree ( ) Neutral ( ) Agree ( ) Strongly agree ( )

1.6. If I receive guidance on preventing breast cancer, I would be willing to incorporate it into my life.
Strongly disagree ( ) Disagree ( ) Neutral ( ) Agree ( ) Strongly agree ( )

1.7. If I experience abnormal breast symptoms, I would promptly seek medical attention.
Strongly disagree ( ) Disagree ( ) Neutral ( ) Agree ( ) Strongly agree ( )

1.8. I feel embarrassed if I have a breast disease.
Strongly disagree ( ) Disagree ( ) Neutral ( ) Agree ( ) Strongly agree ( )

1.9. I believe regular breast health examinations are necessary.
Strongly disagree ( ) Disagree ( ) Neutral ( ) Agree ( ) Strongly agree ( )

Part Two: This section is a survey regarding your understanding of breast-related knowledge.

2.1. Regarding female breasts, which of the following statements do you consider to be correct?
A. Female breasts generally have 15-20 lobes on each side.
B. When there is too little breast tissue hyperplasia or excessive involution, the "stubborn molecules" inside will accumulate more, posing a risk to health.
C. Even if occasional palpable nodules are found in the breasts, it is a normal phenomenon and does not need to be taken seriously.
D. Breast tissue undergoes proliferation and involution in accordance with the menstrual cycle.

2.2. As women age and estrogen secretion decreases, breast tissue degenerates. At this time, breast hyperplasia will:
A. Gradually worsen.
B. Slowly improve.

2.3. Which of the following statements do you believe can prevent breast cancer?
A. Intake of a large amount of dietary fiber whenever possible.
B. Regulating emotions and avoiding negative emotions such as anxiety and depression.
C. Enhancing physical fitness through excessive exercise beyond the body's capacity.
D. Reducing staying up late to avoid endocrine system disorders.

2.4. Regarding breast cancer, which of the following statements do you consider to be correct?
A. The levels of estrogen in the female body are generally stable and do not fluctuate.
B. Fibroadenosis of the breast is essentially a disruption of normal breast structure.
C. The probability of breast hyperplasia developing into breast cancer is very high, and we need to be very cautious.
D. The probability of malignant transformation into breast cancer is higher in patients with a family history of breast cancer.

Reminder: Next is our educational video section. Please adjust the volume and, if necessary, wear headphones to ensure a suitable watching experience. Once you are ready to watch the video, proceed to the next page. Thank you for your cooperation!

☑I have adjusted to a suitable state for watching the video. You can proceed to the next page.

Reminder: Please watch our educational video. (The full-screen button is in the bottom right corner.)

Video watching

Reminder: Thank you for watching our educational video. Next is the post-assessment section. Please answer truthfully and follow the instructions to submit. Thank you for your cooperation!

Part One: This section is a survey about your personal information.
1.1 Gender:
Male ( ) Female ( )

1.2 Age (in years):

1.3 Place of origin (Province + City):

1.4 Marital status:
Unmarried ( ) Married ( ) Divorced ( ) Widowed ( ) Unable to answer ( )

1.5 Occupation:
Student ( )
Worker ( )
Self-employed ( )
Civil servant ( )
Unemployed ( )
Other (please specify: _________)

1.6 Current highest level of education:
No formal education ( ) Primary school ( ) Junior high school ( ) Vocational high school ( ) General high school ( ) Technical school ( ) Junior college ( ) Bachelor's degree ( ) Graduate or above ( ) Other (please specify: _________)

1.7 Your level of interest in the topic of "breast hyperplasia":
Very low ( ) Somewhat low ( ) Neutral ( ) Somewhat high ( ) Very high ( )

1.8 How often do you use short videos to learn about breast health-related knowledge?
Very rarely ( ) Occasionally ( ) Neutral ( ) Frequently ( ) Very frequently ( )

Part Two: This section is a survey about your attitude towards the video.
2.1 Regarding this video, I:
Strongly dislike it ( ) Somewhat dislike it ( ) Neutral ( ) Somewhat like it ( ) Strongly like it ( )

2.2 While watching this video, my attention was highly focused:
Strongly disagree ( ) Somewhat disagree ( ) Neutral ( ) Somewhat agree ( ) Strongly agree ( )

2.3 After watching this video, I believe my level of understanding is:
Very low ( ) Somewhat low ( ) Neutral ( ) Somewhat high ( ) Very high ( )

2.4 Watching this video has increased my understanding of breasts and breast diseases:
Strongly disagree ( ) Somewhat disagree ( ) Neutral ( ) Somewhat agree ( ) Strongly agree ( )

2.5 Regarding the educational approach used in this short video, I:
Strongly dislike it ( ) Somewhat dislike it ( ) Neutral ( ) Somewhat like it ( ) Strongly like it ( )

2.6 I believe the knowledge about breasts mentioned in this video is reliable and accurate:
Strongly disagree ( ) Somewhat disagree ( ) Neutral ( ) Somewhat agree ( ) Strongly agree ( )

2.7 While watching the video, I felt satisfied because I gained new knowledge:
Strongly disagree ( ) Somewhat disagree ( ) Neutral ( ) Somewhat agree ( ) Strongly agree ( )

2.8 The video's warning about the risk of developing breast diseases has triggered negative emotions in me (such as worry, panic, etc.):
Strongly disagree ( ) Somewhat disagree ( ) Neutral ( ) Somewhat agree ( ) Strongly agree ( )

2.9 After watching the video, I am likely to share it with people around me:
Strongly disagree ( ) Somewhat disagree ( ) Neutral ( ) Somewhat agree ( ) Strongly agree ( )

2.10 After watching the video, I would consider applying the knowledge I learned to my daily life:
Strongly disagree ( ) Somewhat disagree ( ) Neutral ( ) Somewhat agree ( ) Strongly agree ( )

2.11 After watching the video, my attitude towards breast health education has changed:
Strongly disagree ( ) Somewhat disagree ( ) Neutral ( ) Somewhat agree ( ) Strongly agree ( )

2.12 For this question, as a test, please select "Somewhat disagree":
Strongly disagree ( ) Somewhat disagree ( ) Neutral ( ) Somewhat agree ( ) Strongly agree ( )

Part Three: This section is a survey about your understanding of the knowledge related to breast cancer mentioned in the video.

3.1 Regarding female breasts, which of the following statements do you believe are correct?
A. A normal adult female breast usually consists of a total of 15-20 lobes.
B. When there is excessive proliferation or insufficient involution of breast tissue, the "stubborn molecules" inside will accumulate, posing a health risk.
C. If a palpable lump-like structure is found in the breast, prompt medical attention should be sought.
D. Breast proliferation and involution are generally stable and do not exhibit significant fluctuations.

3.2 As women age, with a decrease in estrogen secretion and degeneration of breast tissue, what happens to breast hyperplasia?
A. It gradually worsens.
B. It slowly improves.

3.3 Which of the following measures do you believe can prevent breast cancer?
A. Consuming an adequate amount of dietary fiber and avoiding fried foods.
B. Regulating emotions and avoiding negative emotions such as anxiety and depression.
C. Enhancing physical fitness through excessive exercise beyond the body's capacity.
D. Preventing breast cancer by affecting estrogen secretion through staying up late.

3.4 Regarding breast cancer, which of the following statements do you believe are correct?
A. Estrogen levels in the female body fluctuate cyclically with the menstrual cycle.
B. Lobular hyperplasia of the breast significantly increases the risk of breast cancer.
C. The majority of clinically observed breast hyperplasia is benign, but it still significantly increases the incidence of breast cancer.
D. When there is a family history of breast cancer in patients with breast hyperplasia, special attention should be paid to the possibility of malignant transformation into breast cancer.

Part Four: This section is a survey about your attitudes towards various elements in the video.
4.1 What attracted you in this video? (Multiple selections allowed)
A. Expert doctors explaining breast knowledge on camera.
B. The relevance of the topic to daily life.
C. The provision of professional research data in the video.
D. Engaging and interesting animations in the video.
E. Other (please specify: _____)
F. None of the above attracted me.

4.2 What is your opinion regarding the doctor's attire in the video, whether a white coat or casual clothing is preferable?
A. White coat is preferable (Proceed to 4.3)
B. Casual clothing is preferable (Proceed to 4.4)
C. It doesn't matter (Proceed to 4.5)
D. Other (please specify: _____)

4.3 Why do you prefer the doctor to wear a white coat? (Multiple selections allowed)
A. Wearing a white coat aligns with the doctor's image and enhances professionalism.
B. It's difficult to recognize the doctor's identity in casual clothing, and they might be mistaken for non-professionals.
C. The doctor wearing a white coat makes me have more trust in the medical knowledge mentioned in the video.
D. The doctor's presentation in the video is serious, and formal attire like a white coat is appropriate.
E. Other (please specify: _____)

4.4 Why do you prefer the doctor to wear casual clothing? (Multiple selections allowed)

A. The doctor appearing in casual clothing makes me feel more familiar and receptive to the video content.
B. The white coat reminds me of various dangerous diseases, making me feel anxious and uneasy.
C. The doctor's unique style is better showcased in casual clothing, leaving a deeper impression about the video content.
D. The doctor is presenting knowledge to the audience, not working in a hospital, so wearing a white coat is unnecessary and meaningless.
E. Other (please specify: _____)

4.5 Why do you think the doctor's attire doesn't matter? (Multiple selections allowed)
A. I focus on the video content and disregard the doctor's attire.
B. I believe the doctor's attire won't significantly impact my viewing experience of the video.
C. I usually don't pay much attention to the attire of the presenter in educational videos.
D. Other (please specify: _____)

4.6 What improvements do you think this video needs? (Optional)

_____

THE END. Thank you very much!
