# Supplementary material for: Online short videos promoting public breast cancer literacy: a pretest-posttest control group trial on efficiency, attitude, and influencing factors
Source: Front Public Health. 2023 Jun 15;11:1198780. doi: 10.3389/fpubh.2023.1198780 (PMC10310936; doi:10.3389/fpubh.2023.1198780)
Supplement: Supplementary file 2 [file Data_Sheet_2.PDF]

Questionnaire for Video 2

Thank you for participating in this survey! This anonymous survey was initiated by the "Beauty in the Bosom" video team to explore the influencing factors of science popularization through short video dissemination, to promote better science popularization activities. We are committed to creating professional and authoritative short videos on breast knowledge popularization. The team will keep your answers confidential and use them only for research and academic purposes. The questionnaire includes a pre-test, video, and post-test. Please answer truthfully and submit as instructed. Thank you for your cooperation!

Part One: This section is a survey about your knowledge and attitude towards breast diseases.

1.1. I have a good understanding of breast diseases.
Strongly disagree ( ) Disagree ( ) Neutral ( ) Agree ( ) Strongly agree ( )

1.2. I am willing to learn about breast disease-related knowledge.
Strongly disagree ( ) Disagree ( ) Neutral ( ) Agree ( ) Strongly agree ( )

1.3. I believe that watching educational videos about breast diseases helps improve awareness of one's own health.
Strongly disagree ( ) Disagree ( ) Neutral ( ) Agree ( ) Strongly agree ( )

1.4. I believe that my breast health is good.
Strongly disagree ( ) Disagree ( ) Neutral ( ) Agree ( ) Strongly agree ( )

1.5. I am concerned about developing breast cancer in the future.
Strongly disagree ( ) Disagree ( ) Neutral ( ) Agree ( ) Strongly agree ( )

1.6. If I receive guidance on preventing breast cancer, I would be willing to incorporate it into my life.
Strongly disagree ( ) Disagree ( ) Neutral ( ) Agree ( ) Strongly agree ( )

1.7. If I experience abnormal breast symptoms, I would promptly seek medical attention.
Strongly disagree ( ) Disagree ( ) Neutral ( ) Agree ( ) Strongly agree ( )

1.8. I feel embarrassed if I have a breast disease.
Strongly disagree ( ) Disagree ( ) Neutral ( ) Agree ( ) Strongly agree ( )

1.9. I believe regular breast health examinations are necessary.
Strongly disagree ( ) Disagree ( ) Neutral ( ) Agree ( ) Strongly agree ( )

Part Two: This section is a survey about your understanding of breast-related knowledge.

2.1 If someone in your immediate family (same parents) has breast cancer, your probability of developing breast cancer will

A. Significantly increase compared to the general population

B. Slightly higher than the general population

C. Have no difference compared to the general population

D. Slightly lower than the general population

2.2 The more cases of breast cancer there are among your parents and children, the probability of you developing breast cancer will

A. Increase to some extent

B. Remain unchanged

C. Decrease to some extent

2.3 Regarding BRCA1/2 gene mutations, which of the following statements is correct?

A. Males with BRCA1/2 gene mutations should be aware of the risk of prostate cancer.

B. There is no significant relationship between BRCA1/2 gene mutations and breast cancer.

C. Females with BRCA1/2 gene mutations should be aware of the risk of ovarian cancer.

D. BRCA1/2 gene mutations do not affect the genetics of offspring.

Reminder: Next is our educational video section. Please adjust the volume and, if necessary, wear headphones to ensure a suitable watching experience. Once you are ready to watch the video, proceed to the next page. Thank you for your cooperation!

☑I have adjusted to a suitable state for watching the video. You can proceed to the next page.

Reminder: Please watch our educational video. (The full-screen button is in the bottom right corner.)

Video watching

Reminder: Thank you for watching our educational video. Next is the post-assessment section. Please answer truthfully and follow the instructions to submit. Thank you for your cooperation!

Part One: This section is a survey about your personal information.

1.1 Gender:

Male ( ) Female ( )

1.2 Age (in years):

1.3 Place of origin (Province + City):

1.4 Marital status:

Unmarried ( ) Married ( ) Divorced ( ) Widowed ( ) Unable to answer ( )

1.5 Occupation:
Student ( )
Worker ( )
Self-employed ( )
Civil servant ( )
Unemployed ( )
Other (please specify: _________)

1.6 Current highest level of education:
No formal education ( ) Primary school ( ) Junior high school ( ) Vocational high school ( ) General high school ( ) Technical school ( ) Junior college ( ) Bachelor's degree ( ) Graduate or above ( ) Other (please specify: _________)

1.7 How would you rate your current breast health condition?
Very unhealthy ( ) Moderately unhealthy ( ) Average ( ) Moderately healthy ( ) Very healthy ( )

1.8 How often do you use short videos to learn about breast health-related knowledge?
Very rarely ( ) Occasionally ( ) Neutral ( ) Frequently ( ) Very frequently ( )

Part Two: This section is a survey about your attitude towards the video.
2.1 Regarding this video, I:
Strongly dislike it ( ) Somewhat dislike it ( ) Neutral ( ) Somewhat like it ( ) Strongly like it ( )

2.2 While watching this video, my attention was highly focused:
Strongly disagree ( ) Somewhat disagree ( ) Neutral ( ) Somewhat agree ( ) Strongly agree ( )

2.3 After watching this video, I believe my level of understanding is:
Very low ( ) Somewhat low ( ) Neutral ( ) Somewhat high ( ) Very high ( )

2.4 Watching this video has increased my understanding of breasts and breast diseases:
Strongly disagree ( ) Somewhat disagree ( ) Neutral ( ) Somewhat agree ( ) Strongly agree ( )

2.5 Regarding the educational approach used in this short video, I:
Strongly dislike it ( ) Somewhat dislike it ( ) Neutral ( ) Somewhat like it ( ) Strongly like it ( )

2.6 I believe the knowledge about breasts mentioned in this video is reliable and accurate:
Strongly disagree ( ) Somewhat disagree ( ) Neutral ( ) Somewhat agree ( ) Strongly agree ( )

2.7 While watching the video, I felt satisfied because I gained new knowledge:
Strongly disagree ( ) Somewhat disagree ( ) Neutral ( ) Somewhat agree ( ) Strongly agree ( )

2.8 The video's warning about the risk of developing breast diseases has triggered negative emotions in me (such as worry, panic, etc.):
Strongly disagree ( ) Somewhat disagree ( ) Neutral ( ) Somewhat agree ( ) Strongly agree ( )

2.9 After watching the video, I am likely to share it with people around me:
Strongly disagree ( ) Somewhat disagree ( ) Neutral ( ) Somewhat agree ( ) Strongly agree ( )

2.10 After watching the video, I would consider applying the knowledge I learned to my daily life:
Strongly disagree ( ) Somewhat disagree ( ) Neutral ( ) Somewhat agree ( ) Strongly agree ( )

2.11 After watching the video, my attitude towards breast health education has changed:
Strongly disagree ( ) Somewhat disagree ( ) Neutral ( ) Somewhat agree ( ) Strongly agree ( )

2.12 For this question, as a test, please select "Somewhat disagree":
Strongly disagree ( ) Somewhat disagree ( ) Neutral ( ) Somewhat agree ( ) Strongly agree ( )

Part Three: This section is a survey about your understanding of the breast cancer-related knowledge mentioned in the video.

3.1 If your parents have breast cancer, the probability of you developing breast cancer will be_____

A. Significantly higher than the general population
B. Slightly higher than the general population
C. No different from the general population
D. Slightly lower than the general population

3.2 If one sister has breast cancer, the risk for an individual is approximately _____ times of the general population. If two sisters have breast cancer, the risk is approximately _____ times of the general population.

A. 3, 7
B. 30, 70
C. 7, 3
D. 70, 30

3.3 Regarding BRCA1/2 gene mutations, which of the following statements is correct?

A. Males are not affected by BRCA1/2 gene mutations.
B. It is a susceptibility gene for breast cancer.
C. Females with BRCA1/2 gene mutations need to pay attention to breast cancer rather than ovarian cancer.
D. Lesions caused by BRCA1/2 gene mutations often exhibit familial clustering and hereditary characteristics.

3.4 Among the following viewpoints or measures related to breast cancer, which one is correct?

A. Breast cancer has multiple risk factors, and genetics is just one of them.
B. Even if there are high-risk individuals for breast cancer in the family, excessive worry is unnecessary, and genetic testing is not necessary.
C. BRCA1/2 gene mutations do not necessarily have serious consequences, so breast cancer screening is not necessary.
D. Prophylactic mastectomy can reduce the risk of cancer to some extent, but it has not yet received unanimous recognition from guideline experts.

Part Four: This section is a survey about your attitude towards elements in the video.
4.1 What attracted you in this video? (Multiple choices allowed)
A. Professional doctors explaining breast knowledge
B. The topic being relevant to daily life
C. Providing professional research data in the video
D. Engaging and interesting animations in the video
E. Other (Please specify _____)
F. Nothing attracted me

4.2 Do you think this video needs background music?
A. Yes (Proceed to 4.3)
B. No (Proceed to 4.4)

4.3 Why do you think this video needs background music?
A. Background music can create a harmonious atmosphere and alleviate my fear of breast cancer.
B. Background music can enhance my interest in watching the video.
C. Background music can create a pleasant mood for me.
D. Other (Please specify _____)

4.4 Why do you think this video does not need background music?
A. I believe that background music has a minimal impact on the viewing experience.
B. I prefer to enjoy the tranquility of a video without background music.
C. I'm concerned that background music might distract my attention.
D. Other (Please specify _____)

4.5 What other aspects do you think need improvement in this video? (Optional)

_____

THE END. Thank you very much!
