# Supplementary material for: Online short videos promoting public breast cancer literacy: a pretest-posttest control group trial on efficiency, attitude, and influencing factors
Source: Front Public Health. 2023 Jun 15;11:1198780. doi: 10.3389/fpubh.2023.1198780 (PMC10310936; doi:10.3389/fpubh.2023.1198780)
Supplement: Supplementary file 3 [file Data_Sheet_3.PDF]

Questionnaire for Video 3

Thank you for participating in this survey! This anonymous survey was initiated by the "Beauty in the Bosom" video team to explore the influencing factors of science popularization through short video dissemination, to promote better science popularization activities. We are committed to creating professional and authoritative short videos on breast knowledge popularization. The team will keep your answers confidential and use them only for research and academic purposes. The questionnaire includes a pre-test, video, and post-test. Please answer truthfully and submit as instructed. Thank you for your cooperation!

Part One: This section is a survey about your knowledge and attitude towards breast diseases.

1.1. I have a good understanding of breast diseases.
Strongly disagree ( ) Disagree ( ) Neutral ( ) Agree ( ) Strongly agree ( )

1.2. I am willing to learn about breast disease-related knowledge.
Strongly disagree ( ) Disagree ( ) Neutral ( ) Agree ( ) Strongly agree ( )

1.3. I believe that watching educational videos about breast diseases helps improve awareness of one's own health.
Strongly disagree ( ) Disagree ( ) Neutral ( ) Agree ( ) Strongly agree ( )

1.4. I believe that my breast health is good.
Strongly disagree ( ) Disagree ( ) Neutral ( ) Agree ( ) Strongly agree ( )

1.5. I am concerned about developing breast cancer in the future.
Strongly disagree ( ) Disagree ( ) Neutral ( ) Agree ( ) Strongly agree ( )

1.6. If I receive guidance on preventing breast cancer, I would be willing to incorporate it into my life.
Strongly disagree ( ) Disagree ( ) Neutral ( ) Agree ( ) Strongly agree ( )

1.7. If I experience abnormal breast symptoms, I would promptly seek medical attention.
Strongly disagree ( ) Disagree ( ) Neutral ( ) Agree ( ) Strongly agree ( )

1.8. I feel embarrassed if I have a breast disease.
Strongly disagree ( ) Disagree ( ) Neutral ( ) Agree ( ) Strongly agree ( )

1.9. I believe regular breast health examinations are necessary.
Strongly disagree ( ) Disagree ( ) Neutral ( ) Agree ( ) Strongly agree ( )

Part Two: This section is a survey regarding your understanding of breast-related knowledge.

2.1 Which of the following factors do you think does not reduce the risk of developing breast cancer?
A. Mother giving birth to children
B. Oral contraceptives with high-dose estrogen
C. Breastfeeding
D. None of the above

2.2 Regarding estrogen, which of the following statements do you think is correct?
A. Excessive estrogen can lead to uncontrolled growth of breast epithelial cells.
B. Moderate levels of estrogen are beneficial to the human body.
C. Normal individuals rely solely on endogenous synthesis of estrogen and do not intake it from external sources.
D. Estrogen levels in the body are relatively low during pregnancy and lactation.

2.3 Regarding breast cancer, which of the following statements do you think is correct?
A. Marriage and childbirth cannot completely prevent breast cancer.
B. The more children a woman has, the higher the probability of developing breast cancer.
C. It is currently uncertain whether the risk of breast cancer is related to the number of breastfeeding instances.
D. Reducing the frequency of breast tissue exposure to high estrogen levels will lower the incidence of breast cancer.

2.4 Regarding breast tissue cells, which of the following statements do you think is correct?
A. Pregnancy and lactation promote the maturation of breast tissue cells.
B. During pregnancy and lactation, breast tissue cells become less resistant to carcinogens.
C. Estrogen can stimulate the growth and proliferation of breast epithelial cells.
D. When breast epithelial cells undergo abnormal proliferation, they are promptly dealt with by the body's immune system and do not lead to breast cancer.

Reminder: Next is our educational video section. Please adjust the volume and, if necessary, wear headphones to ensure a suitable watching experience. Once you are ready to watch the video, proceed to the next page. Thank you for your cooperation!

☑I have adjusted to a suitable state for watching the video. You can proceed to the next page.

Reminder: Please watch our educational video. (The full-screen button is in the bottom right corner.)

Video watching

Reminder: Thank you for watching our educational video. Next is the post-assessment section. Please answer truthfully and follow the instructions to submit. Thank you for your cooperation!

Part One: This section is a survey about your personal information.
1.1 Gender:
Male ( ) Female ( )

1.2 Age (in years):

1.3 Place of origin (Province + City):

1.4 Marital status:
Unmarried ( ) Married ( ) Divorced ( ) Widowed ( ) Unable to answer ( )

1.5 Occupation:
Student ( )
Worker ( )
Self-employed ( )
Civil servant ( )
Unemployed ( )
Other (please specify: _________)

1.6 Current highest level of education:
No formal education ( ) Primary school ( ) Junior high school ( ) Vocational high school ( ) General high school ( ) Technical school ( ) Junior college ( ) Bachelor's degree ( ) Graduate or above ( ) Other (please specify: _________)

1.7 How interested are you in the topic of "Marriage, Childbirth, and Breast Cancer"?
Very low ( ) Somewhat low ( ) Neutral ( ) Somewhat high ( ) Very high ( )

1.8 How often do you use short videos to learn about breast health-related knowledge?
Very rarely ( ) Occasionally ( ) Neutral ( ) Frequently ( ) Very frequently ( )

Part Two: This section is a survey about your attitude towards the video.
2.1 Regarding this video, I:
Strongly dislike it ( ) Somewhat dislike it ( ) Neutral ( ) Somewhat like it ( ) Strongly like it ( )

2.2 While watching this video, my attention was highly focused:
Strongly disagree ( ) Somewhat disagree ( ) Neutral ( ) Somewhat agree ( ) Strongly agree ( )

2.3 After watching this video, I believe my level of understanding is:
Very low ( ) Somewhat low ( ) Neutral ( ) Somewhat high ( ) Very high ( )

2.4 Watching this video has increased my understanding of breasts and breast diseases:
Strongly disagree ( ) Somewhat disagree ( ) Neutral ( ) Somewhat agree ( ) Strongly agree ( )

2.5 Regarding the educational approach used in this short video, I:
Strongly dislike it ( ) Somewhat dislike it ( ) Neutral ( ) Somewhat like it ( ) Strongly like it ( )

2.6 I believe the knowledge about breasts mentioned in this video is reliable and accurate:
Strongly disagree ( ) Somewhat disagree ( ) Neutral ( ) Somewhat agree ( ) Strongly agree ( )

2.7 While watching the video, I felt satisfied because I gained new knowledge:
Strongly disagree ( ) Somewhat disagree ( ) Neutral ( ) Somewhat agree ( ) Strongly agree ( )

2.8 The video's warning about the risk of developing breast diseases has triggered negative emotions in me (such as worry, panic, etc.):
Strongly disagree ( ) Somewhat disagree ( ) Neutral ( ) Somewhat agree ( ) Strongly agree ( )

2.9 After watching the video, I am likely to share it with people around me:
Strongly disagree ( ) Somewhat disagree ( ) Neutral ( ) Somewhat agree ( ) Strongly agree ( )

2.10 After watching the video, I would consider applying the knowledge I learned to my daily life:
Strongly disagree ( ) Somewhat disagree ( ) Neutral ( ) Somewhat agree ( ) Strongly agree ( )

2.11 After watching the video, my attitude towards breast health education has changed:
Strongly disagree ( ) Somewhat disagree ( ) Neutral ( ) Somewhat agree ( ) Strongly agree ( )

2.12 For this question, as a test, please select "Somewhat disagree":
Strongly disagree ( ) Somewhat disagree ( ) Neutral ( ) Somewhat agree ( ) Strongly agree ( )

Part Three: This section is a survey about your understanding of the knowledge related to breast cancer mentioned in the video.

3.1 After watching the video, which of the following factors do you think are protective against breast cancer?
A. Mother giving birth to children
B. Oral contraceptives with high-dose estrogen
C. Using animal milk instead of breastfeeding
D. All of the above

3.2 Regarding estrogen mentioned in the video, which of the following statements do you think is correct?
A. Excessive estrogen can lead to uncontrolled growth of a small number of breast epithelial cells.
B. Estrogen is harmful to the human body, and the less, the better.
C. Normal individuals can intake estrogen from external sources in addition to endogenous synthesis.
D. Estrogen levels in the body are relatively high during pregnancy and lactation.

3.3 After watching the video, which of the following statements do you think is correct?
A. Epidemiological studies suggest that lifelong unmarried individuals have a higher probability of developing breast cancer compared to married individuals.
B. The probability of developing breast cancer does not change with the number of children a woman has.
C. A higher frequency of breastfeeding may potentially reduce the probability of developing breast cancer.
D. When breast tissue is frequently exposed to high estrogen levels, the incidence of breast cancer will significantly decrease.

3.4 Regarding breast tissue cells mentioned in the video, which of the following statements do you think is correct?
A. Pregnancy and lactation cause mature breast tissue cells to shed, promoting cell proliferation and the formation of more immature breast tissue cells.
B. During pregnancy and lactation, breast tissue cells become more resistant to carcinogenic factors.
C. Estrogen can stimulate the growth and proliferation of all breast tissue cells.
D. Abnormal proliferation of breast epithelial cells may eventually lead to breast cancer.

Part Four: This section is a survey about your attitude towards elements in the video.
4.1 What attracted me in this video is (multiple choices possible):
A. A professional doctor explaining breast knowledge on screen.
B. The relevance of the topic to daily life.
C. The presentation of professional research data in the video.
D. The engaging and interesting animations in the video.
E. Other (please specify: _____).
F. Nothing attracted me.

4.2 Do you think it is necessary to have a progress bar indicating the various sections of the video at the bottom?
A. Yes (Go to 4.3)
B. No (Go to 4.4)

4.3 Why do you think this progress bar is necessary?
A. It indicates the main content of the video.
B. It helps deepen my understanding of the video content.
C. It makes me find it novel and interesting.
D. It gives me a psychological expectation of the duration of each section.
E. Other (please specify: _____).

4.4 Why do you think this progress bar is not necessary?
A. I don't care too much about the progress when watching short videos.
B. It distracts me and affects my normal viewing and thinking of the video content.
C. The design of the progress bar itself is not well done (e.g., position, text content, etc.).

D. Other (please specify: _____).

4.5 In your opinion, what other aspects of this video need improvement? (Optional)

_____

THE END. Thank you very much!
